# Review of the leafhopper genus *Eurhadina* Haupt, 1929 from China, with description of two new species (Hemiptera, Cicadellidae, Typhlocybinae, Typhlocybini)

**DOI:** 10.3897/zookeys.1284.198471

**Published:** 2026-07-09

**Authors:** Xiaolong Huang, Jun Wang, Liuhong Tang, Michael D. Webb, Yani Duan

**Affiliations:** 1 Anhui Province Key Laboratory of Integrated Pest Management on Crops, Key Laboratory of Biology and Sustainable Management of Plant Diseases and Pests of Anhui Higher Education Institutes, School of Plant Protection, Anhui Agricultural University, Hefei, Anhui 230036, China Anhui Province Key Laboratory of Integrated Pest Management on Crops, Key Laboratory of Biology and Sustainable Management of Plant Diseases and Pests of Anhui Higher Education Institutes, School of Plant Protection, Anhui Agricultural University Hefei China; 2 Department of Life Sciences, The Natural History Museum (retired), London, SW7 5BD, UK Department of Life Sciences, The Natural History Museum London United Kingdom

**Keywords:** Auchenorrhyncha, identification key, morphology, taxonomy

## Abstract

Two new species of the leafhopper genus *Eurhadina* Haupt, 1929 (Typhlocybinae, Typhlocybini) from China are described and illustrated: E. (Eurhadina) cervina Tang, **sp. nov**. and E. (Eurhadina) dashuensis Tang, **sp. nov**. A key to the subgenera of *Eurhadina*, a key to the Chinese species of E. (Eurhadina) Haupt, 1929, and an appendix with a checklist of Chinese *Eurhadina* species are provided. The subgenus and species E. (Zhihadina) sinica Yang & Li, 1991 are treated as *nomen dubia* and *incertae sedis* in Typhlocybinae. Diagnoses of the recognized subgenera are provided, and figures of E. (Singhardina) rubrocorona Cai & Kuoh, 1993 are given for comparison with the nominate subgenus.

## Introduction

Typhlocybini (Hemiptera, Cicadellidae, Typhlocybinae) is one of the largest tribes within the subfamily Typhlocybinae. To date, 96 genera and 971 species of Typhlocybini have been documented worldwide (Dmitriev et al. 2022). The genus *Eurhadina* Haupt, 1929 is a member of the tribe Typhlocybini and is distributed across the Palaearctic and Oriental regions and currently comprises three subgenera: E. (Eurhadina) Haupt, 1929, E. (Singhardina) Mahmood, 1967, and E. (Zhihadina) Yang & Li, 1991. The last-mentioned subgenus, with E. (Z.) sinica Yang & Li, 1991 as its type species, is treated here as a nomen dubium and placed as *incertae sedis* in Typhlocybinae due to conflicting descriptions and the loss of type specimens, as discussed below. Until now, 99 species of *Eurhadina* have been described worldwide, and China, with 55 species, has the greatest species diversity.

The subgenus E. (Eurhadina) is predominantly distributed in the Palaearctic region and currently comprises 17 recognized species worldwide, of which 14 are recorded from Asia and five from China. This study describes and illustrates two new species belonging to this subgenus, bringing the total number of known Chinese species to seven. In addition, this paper provides a key and diagnoses to the two recognized *Eurhadina* subgenera, a revised identification key to all the known species of the subgenus E. (Eurhadina) from China, and an appendix with a checklist of *Eurhadina* from China.

## Materials and methods

Leafhopper specimens were collected using a sweep net in Anhui Province, China, in 2024. After collection, specimens were sorted and preserved in 90% ethanol for subsequent examination. Body length was measured from the apex of the vertex to the tip of the forewings. Prior to detailed examination, the entire abdomen was dissected and cleared in 10% NaOH to remove soft tissues and muscles (maceration), then rinsed thoroughly with distilled water. The genitalia were mounted on a glass slide with a drop of glycerol for observation and photo documentation, then placed in a multiple-cavity tray for long-term storage. The remaining body parts, excluding the abdomen, were returned to ethanol for preservation.

Identifications were performed based on external morphological characters and male genital structures. Digital images were captured using a Nikon DS-Ri2 camera attached to either a Nikon SMZ 1500 stereomicroscope or a Nikon Eclipse 50i microscope. All photographs were post-processed in Adobe Photoshop 2025 (v. 26.0.0) to remove the background and adjust colour balance and contrast.

Morphological terminology follows Dietrich et al. (2022). The examined material, including the holotypes, is deposited in the School of Plant Protection, Anhui Agricultural University, Hefei, Anhui, China (acronym: **SPPAAU**).

## Taxonomy

**Typhlocybini Kirschbaum, 1868**

### Eurhadina

Taxon classificationAnimaliaHemipteraCicadellidae

Haupt, 1929

10994FF9-5E6F-5B99-B9E8-8F62DDEC9ED5

Eurhadina Haupt, 1929: 1075.

#### Type species.

*Cicada pulchella* Fallén, 1806; by original designation.

#### Diagnosis.

Colouration variable. Forewing apex with transverse streaks; RP vein with one or more black spots or markings. Body flattened. Head equal to or narrower than pronotum; crown usually obtusely rounded anteriorly; face swollen anteriorly or flattened. Forewing with third apical cell usually petiolate; fourth apical cell largest. Hindwing tapered towards apex, with three cross-veins and submarginal vein absent in distal part of wing. Abdominal apodemes usually extending beyond the 4^th^ abdominal segment. Side of male pygofer posteriorly cleft into dorsal and ventral lobes; ventral margin deeply incurved adjacent to the valve. Subgenital plate usually with one stout lateral macroseta. Paramere with apophysis scythe-like; outer margin setose. Connective Y-shaped; stem slender or broad, with medial keel. Aedeagal shaft with one or two pairs of apical processes, sometimes branched.

#### Distribution.

Palaearctic, Afrotropical, and Oriental regions.

#### Remarks.

**Figure 1.**
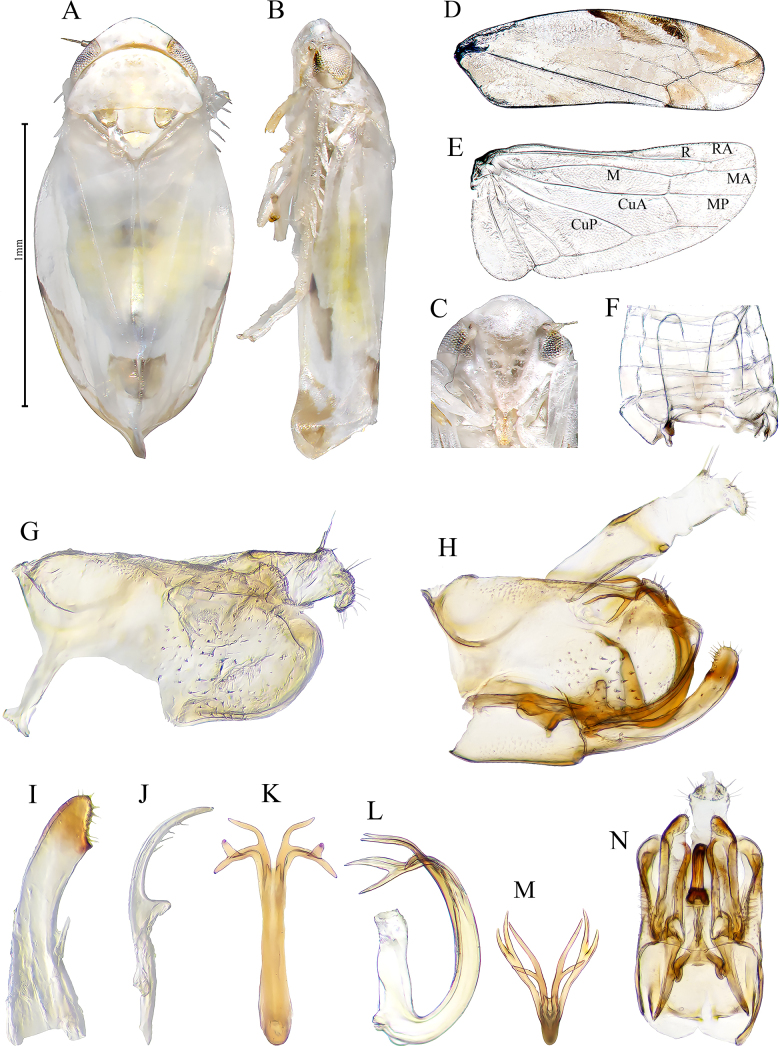
Eurhadina (Eurhadina) cervina Tang, sp. nov., male, holotype. **A**. Habitus, dorsal view; **B**. Habitus, lateral view; **C**. Face; **D**. Forewing; **E**. Hindwing; **F**. Male abdominal apodemes; **G**. Side of male pygofer and anal tube, lateral view; **H**. Male genital capsule and anal tube, lateral view; **I**. Subgenital plate, dorsal view; **J**. Style, dorsal view; **K**. Aedeagus, dorsal view; **L**. Aedeagus, lateral view; **M**. Apical part of aedeagus, dorsal view; **N**. Male genital capsule and anal tube, ventral view.

Ribaut (1936) treated *Eurhadina* as a junior synonym of *Eupteryx* Curtis, 1831, but later, Young (1952) and Dlabola (1958) independently reinstated its generic status. Young (1952) grouped *Eurhadina* together with *Eupterella* DeLong & Ruppel, 1950, *Eupteroidea* Young, 1952, and *Eupteryx* Curtis, 1831 in the *Eupteryx* complex of the tribe Typhlocybini, united by the common character of three subapical cross-veins in the hindwing (Fig. 1E). Subsequently, Dworakowska (1969) also placed *Asymmetropteryx* Dlabola, 1958 and *Wagneripteryx* Dlabola, 1958 in the *Eupteryx* complex and also reviewed the taxonomic history of *Eurhadina*, provided a detailed discussion on its generic diagnostic characters, included *Singhardina* as its subgenus, and presented a key to the two included subgenera and their species. Here we show a previously undescribed feature in the side of male pygofer; that is, the ventral margin adjacent to the valve is deeply incurved (Fig. 1G) and connected to the valve by membrane.

Lastly, Yang and Li (1991) described a third subgenus, E. (Zhihadina), based on a single new species, E. (Z.) sinica from China. However, the description is based on two different species, one based on the external figures belonging to *Eurhadina* (Typhlocybini) and the other, based on the male genitalia figures, belonging to Arboridia (Arborifera) Sohi & Sandhu, 1971 (Erythroneurini), and the diagnosis of this subgenus reiterates this situation in the following way: “Externally very similar to the subgenus *Eurhadina*, but male genitalia distinctly different” (Yang and Li 1991: 28, translated from Chinese). To rectify this situation, one option would be to synonymise E. (Z.) sinica with another species, thereby making the species invalid. However, the most similar species based on colour marking in the same publication, E. (S.) centralis Yang & Li, 1991, and which was also collected on the same date and in the same place as E. (Z.) sinica, lacks the forewing petiolate third apical cell present in the latter. Another species in the same publication, with the same data, E. (S.) flavostriata Yang & Li, 1991 but incorrectly referred to as E. (S.) flavivittiata Yang & Li, 1991 in the figure legend, has different colour marking. Synonymising E. (Z.) sinica with another species, based on the original male genitalia figures, is also not possible. The shape of its aedeagus comes close to A. (A.) sohii Dworakowska, 1980, A. (A.) vinealis (Ahmed, 1970), and A. (A.) viniferata Sohi & Sandhu, 1971, but there are some differences (Yuehua Song pers. comm.). The type specimens of all species described by Yang and Li (1991), including E. (Z.) sinica, were stated to have been deposited in the China Agricultural University, but efforts to find them there were unsuccessful. Based on the above information, it is probably best to consider both the subgenus and species E. (Z.) sinica as *nomen dubia*, that is, of uncertain identity, and *incertae sedis* of uncertain placement.

We provide here a key to the two recognized subgenera, revised diagnoses, a revised key to the Chinese E. (Eurhadina), and an appendix with a checklist of Chinese *Eurhadina*.

##### Key to the subgenera of *Eurhadina*

**Table:** 

1	Upper part of face swollen in lateral view (Fig. 1B); forewing with third apical cell with a basal stem (petiolate) (Fig. 1D); side of male pygofer not posteriorly sclerotized (Fig. 1G); subgenital plate club-like, with a subapical process (Fig. 1I)	**E. (Eurhadina) Haupt, 1929**
–	Upper part of face flattened in lateral view (Fig. 3B); forewing with third apical cell with or without a basal stem; side of male pygofer posteriorly sclerotized (Fig. 3H); subgenital plate strongly distally tapered to slightly swollen apex, with the latter bearing small, spine-like setae, without a subapical process (Fig. 3I)	**E. (Singhardina) Mahmood, 1967**

### Eurhadina (Eurhadina)

Taxon classificationAnimaliaHemipteraCicadellidae

Haupt, 1929

DCD3CF04-8924-5935-BEB8-8030DC2A078E

Eurhadina Haupt, 1929: 1075.
Cicadella (Eurhadina) : Kloet and Hincks 1945: 56.
Eurhadina (Eurhadina) : Dworakowska 1969: 68 (stat. nov., in key).

#### Diagnosis.

Body wide and flattened, with dull colouration. Upper part of face swollen in lateral view. Crown obtusely rounded and protruding, with medial length distinctly shorter than interocular width; median part of frontoclypeus slightly raised. Forewing with third apical cell petiolate; hindwing with three subapical cross-veins. Side of male pygofer with distinct or indistinct posterior cleft. Subgenital plate club-shaped, with subapical marginal process and a single lateral macroseta; apex with club-like or fine setae.

#### Distribution.

Palaearctic, Afrotropical, and Oriental regions.

#### Remarks.

See above key for differences between this and E. (Singhardina).

##### Key to species of subgenus Eurhadina (Eurhadina) from China

The key is modified from Zhou et al. (2022).

**Table:** 

1	Forewing without a black circular spot on RP vein (Fig. 1D)	**E. (E.) cervina Tang, sp. nov**.
–	Forewing with a black circular spot on RP vein (Fig. 2E)	**2**
2	Aedeagal shaft with unbranched apical processes	**E. (E.) fujiana Yang & Li, 1991**
–	Aedeagal shaft with bifurcate apical processes (Fig. 2K, L)	**3**
3	Aedeagal shaft with two pairs of apical processes; both pairs bifurcate	**4**
–	Aedeagal shaft with two pairs of apical processes; one pair bifurcate, the other unbranched (Fig. 2K, L)	**5**
4	Aedeagal shaft with serrations on subapical part of dorsal margin	**E. (E.) pulchella (Fallén, 1806)**
–	Aedeagal shaft without serrations on subapical part of dorsal margin	**E. (E.) japonica Dworakowska, 1971**
5	Dorsal and ventral processes of aedeagal shaft similar in length (Fig. 2K, L)	**6**
–	Dorsal processes of aedeagal shaft significantly shorter than ventral processes	**E. (E.) alba Dworakowska, 1979**
6	Bifurcations of ventral aedeagal processes significantly different in size, with fork relatively narrow (Fig. 2K, L)	**E. (E.) dashuensis Tang, sp. nov**.
–	Bifurcations of ventral aedeagal processes not significantly different in size, with fork relatively wide	**E. (E.) callissima Dworakowska, 1967**

### Eurhadina (Eurhadina) cervina

Taxon classificationAnimaliaHemipteraCicadellidae

Tang sp. nov.

F0060503-F761-59DB-9D8B-7BAD639E8CE3

https://zoobank.org/3EA8FF9D-8487-4B45-B53E-093A83B01EE1

Fig. 1A–N

#### Type material.

***Holotype***: China • ♂; Anhui Prov., Hefei, Dashu Mountain National Forest Park; 31°50'N, 117°14'E; alt. 284 m; 8 Jul. 2024; Bingqing Xie, Liuhong Tang & Baoling Qian leg.; AAU TLH028-C8.

#### Description.

***Measurements***. Length: male 1.5 mm.

***Colouration***. Body colour creamy white. Face slightly paler creamy white. Pronotum pale yellowish white, with a slightly translucent spot beneath each eye. Mesonotum and scutellum pale yellow, with distinct triangular markings (Fig. 1A–C). Forewing with faint brownish markings in distal half (Fig. 1D).

***Morphology***. Body small and flattened. Head slightly narrower than pronotum; crown with anterior margin angularly produced forward; interocular width clearly greater than mid-crown length; median part of frontoclypeus slightly raised and somewhat broad; genae narrow (Fig. 1A–C). Forewing with apical-cell region occupying 1/3 of wing length. Hindwing with three subapical cross-veins (Fig. 1D, E). Abdominal apodemes extending to the intersegmental region between sternites VII and VIII (Fig. 1F).

***Male genitalia***. Side of male pygofer dorso-posterior margin shallowly cleft forming two lobes; upper lobe produced dorsad, its posterior margin bearing several setae; lower lobe strongly sclerotized at ventro-apical angle; ventral margin of side of male pygofer deeply incurved adjacent to valve, with scattered microsetae in lateral view. Anal tube well developed (Fig. 1G, H). Subgenital plate with one stout, long seta arising from outer margin near basal 1/3; apex bearing eight stout, peg-like microsetae; inner margin nearly parallel to outer margin and arcuate (Fig. 1I). Style slender and elongate; apophysis scythe-shaped, with a few fine subapical setae on outer margin (Fig. 1J). Aedeagus with two pairs of apical processes; dorsal processes branched laterad near base; ventral processes branched on apical outer margin; in dorsal view, apex of aedeagus resembling a stag’s antlers (Fig. 1K–M). Connective Y-shaped; central stem thick, long, medially carinate, weakly sclerotized; lateral arms slender and short (Fig. 1N).

#### Etymology.

The specific name is derived from the Latin word “cervi”, meaning “stag” or “deer”, referring to the shape of the aedeagal processes like a stag’s antlers. The specific epithet is treated as an adjective.

#### Distribution.

Known only from the type locality.

#### Remarks.

The new species is morphologically similar to Eurhadina (Eurhadina) japonica Dworakowska, 1971 in general external appearance, but it can be readily distinguished by marked differences in body size and aedeagal structure. The new species has a body length of 1.5 mm, distinctly smaller than the 2.7–3.3 mm body length recorded for E. (E.) japonica. For the aedeagus, the dorsal processes of the aedeagal shaft are branched at both the base and apex in E. (E.) japonica, while in the new species these processes are only branched basally. Furthermore, E. (E.) japonica consistently exhibits a small dark brown circular spot at the midpoint of the RP vein on the forewing, a marking that is entirely absent in the new species.

### Eurhadina (Eurhadina) dashuensis

Taxon classificationAnimaliaHemipteraCicadellidae

Tang sp. nov.

75EF3A37-A496-534C-8BA3-B223EE74E205

https://zoobank.org/14DAA95C-75DF-484B-B3D0-C33EA41F5754

Fig. 2A–N

#### Type material.

***Holotype***: China • ♂; Anhui Prov., Hefei, Dashu Mountain National Forest Park; 31°50'N, 117°14'E; alt. 284 m; 8 Jul. 2024; Bingqing Xie, Liuhong Tang & Baoling Qian leg.; AAU TLH032-D2. ***Paratype***: China • 1♂; same data as for holotype; AAU TLH032-D11.

#### Description.

***Measurements***. Length: male 3.3 mm.

**Figure 2.**
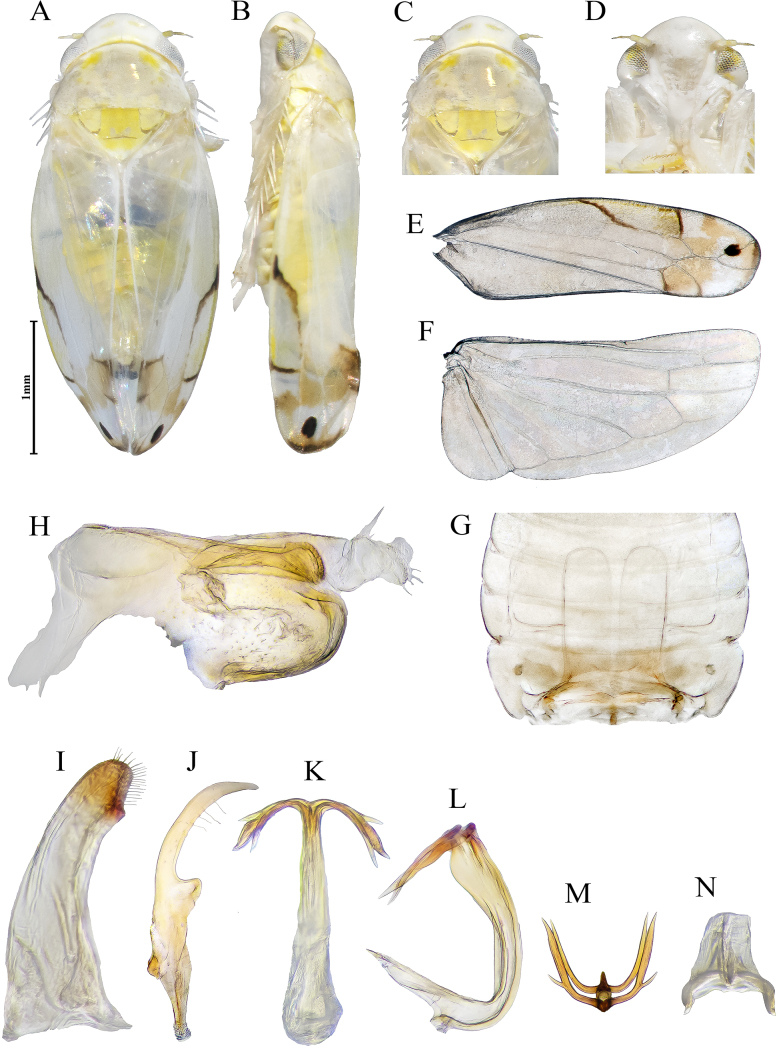
Eurhadina (Eurhadina) dashuensis Tang, sp. nov., male, holotype. **A**. Habitus, dorsal view; **B**. Habitus, lateral view; **C**. Head and thorax, dorsal view; **D**. Face; **E**. Forewing; **F**. Hindwing; **G**. Male abdominal apodemes; **H**. Side of male pygofer and anal tube, lateral view; **I**. Subgenital plate, dorsal view; **J**. Style, dorsal view; **K**. Aedeagus, dorsal view; **L**. Aedeagus, lateral view; **M**. Apical part of aedeagus, dorsal view; **N**. Connective, dorsal view.

***Colouration***. Head and pronotum creamy white; crown submarginally with a pair of light-yellow round spots; compound eyes silvery grey. Pronotum with two symmetrical yellow spots. Mesonotum and scutellum yellow, with distinct triangular markings, its tip creamy white (Fig. 2A–D). Apical-cell region of the forewing with pale-brown markings, and the RP vein with a distinct dark-brown spot at junction of first and second apical cells (Fig. 2E).

***Morphology***. Body flattened. Head narrower than pronotum; crown with anterior margin arched forward; medial length equals half interocular width; median part of frontoclypeus slightly raised and broad. Pronotum with posterior margin slightly emarginate and translucent (Fig. 2A–D). Apical-cell region of the forewing occupying 1/3 of wing length. Hindwing with three subapical cross-veins; marginal vein not reaching CuA vein (Fig. 2E, F). Abdominal apodemes extending to sternite VI (Fig. 2G).

***Male genitalia***. Side of male pygofer with a few minute setae subapically on ventral margin; dorso-posterior margin distinctly cleft at apex with upper lobe bearing several setae and lower lobe apically sclerotized; ventral margin of side of male pygofer deeply incurved adjacent to valve. Anal tube well developed (Fig. 2H). Subgenital plate nearly rectangular, medially emarginate, with one stout, long, subbasal seta and a row of fine setae along apical margin (Fig. 2I). Style slender; apophysis scythe-shaped, with a few fine subapical setae on outer margin (Fig. 2J). Aedeagal shaft robust, slightly subapically swollen; tip evenly C-shaped in lateral view, with two pairs of apical processes, dorsal processes unbranched, the bifurcations of the ventral aedeagal process are markedly unequal in size (Fig. 2K–M). Connective Y-shaped, with a broad median stem whose anterior margin is straight; arms short, slender, laterally arcuate (Fig. 2N).

#### Etymology.

The species is named after Dashu Mountain, where the type locality is located.

#### Distribution.

Known only from the type locality.

#### Remarks.

This species closely resembles Eurhadina (Eurhadina) callissima Dworakowska, 1967 in external morphology and male genitalia. However, it differs from E. (E.) callissima in the ventral process of the aedeagus. In the new species, the bifurcations of the ventral aedeagal process are markedly unequal in size and the fork is relatively narrow, while in E. (E.) callissima the bifurcations are nearly equal in size and the fork is broader.

### Eurhadina (Singhardina)

Taxon classificationAnimaliaHemipteraCicadellidae

Mahmood, 1967

0291833D-7590-58C1-A608-713CCA5CED9E

Singhardina Mahmood, 1967: 32.
Eurhadina (Singhardina) : Sohi and Dworakowska 1983: 188.

#### Type species.

*Singhardina robusta* Mahmood, 1967.

#### Diagnosis.

**Figure 3.**
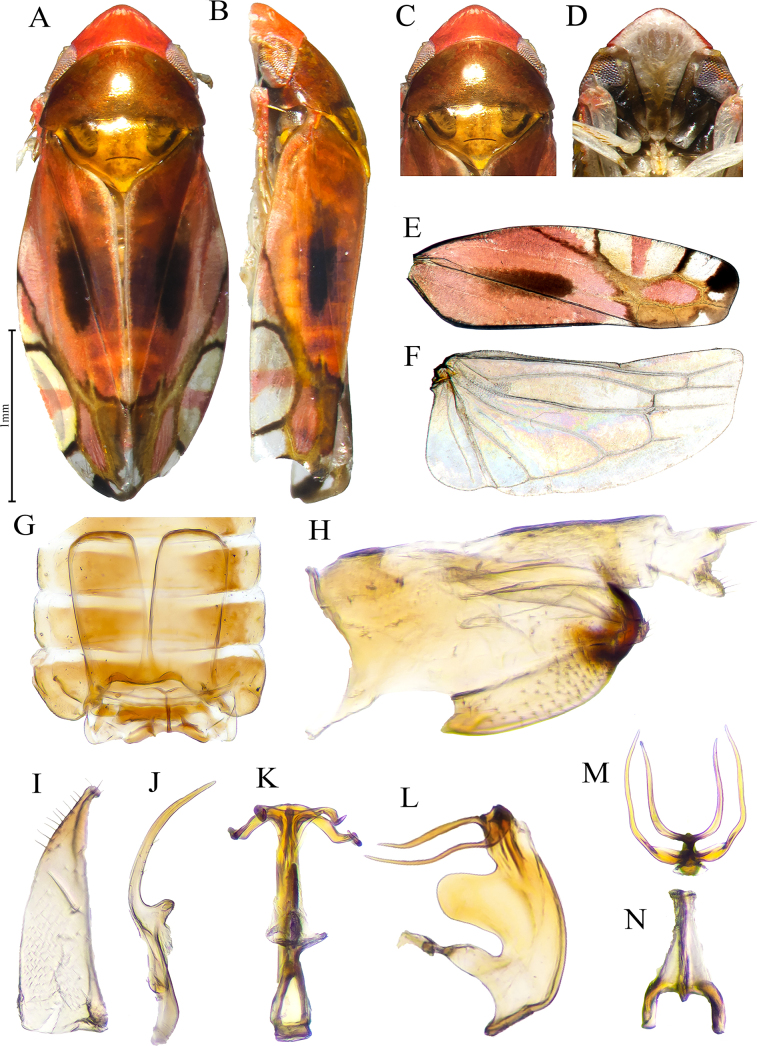
Eurhadina (Singhardina) rubrocorona Cai & Kuoh, 1993, male. **A**. Habitus, dorsal view; **B**. Habitus, lateral view; **C**. Head and thorax, dorsal view; **D**. Face; **E**. Forewing; **F**. Hindwing; **G**. Male abdominal apodemes; **H**. Side of male pygofer and anal tube, lateral view; **I**. Subgenital plate, dorsal view; **J**. Style, dorsal view; **K**. Aedeagus, dorsal view; **L**. Aedeagus, lateral view; **M**. Apical part of aedeagus, dorsal view; **N**. Connective, dorsal view.

Body wide and flattened, with dull to bright colouration. Crown obtusely rounded and protruding, with medial length distinctly shorter than interocular width; face flattened in lateral view (Fig. 3A–D). Forewing with or without third apical cell petiolate. Hindwing with three subapical cross-veins (Fig. 3E, F). Side of male pygofer with or without posterior cleft (Fig. 3H). Subgenital plate strongly distally tapered, with apex expanded; the latter with a few spine-like setae (Fig. 3I).

#### Distribution.

Palaearctic and Oriental regions.

#### Remarks.

See the key above for differences between this and nominate subgenus. Zhou et al. (2022) reviewed the subgenus and provided a key to its species, and below we provide a checklist of all included species from China. We also figure E. (S.) rubrocorona Cai & Kuoh, 1993 for comparison with the nominate subgenus.

## Discussion

This study describes two new species of the subgenus Eurhadina (Eurhadina) from China, increasing the number of known Chinese species in this subgenus from five to seven. The two new species, E. (E.) cervina Tang, sp. nov. and E. (E.) dashuensis Tang, sp. nov., are primarily distinguished by their unique aedeagal characteristics. In E. (E.) cervina Tang, sp. nov., the aedeagal processes are shaped like stag’s antlers, a configuration that has not previously been documented in the genus. In E. (E.) dashuensis Tang, sp. nov., the ventral processes are asymmetrically bifurcated, and this feature differentiates it from the morphologically similar E. (E.) callissima.

Additionally, we report a previously overlooked side of male pygofer character: the ventral margin adjacent to the valve is deeply incurved in the species of *Eurhadina* studied here (Figs 1G, 2H, 3H). This character may serve as a valuable diagnostic feature for the genus, but its occurrence needs to be checked in other species of the genus and related genera.

This study has several limitations, including the lack of molecular and host-plant data, the absence of female specimens for the two new species, and the unresolved taxonomic status of *Zhihadina*. Future research integrating DNA barcoding and host plant records will further clarify species boundaries and evolutionary relationships within the genus *Eurhadina*.

## Supplementary Material

XML Treatment for Eurhadina

XML Treatment for Eurhadina (Eurhadina)

XML Treatment for Eurhadina (Eurhadina) cervina

XML Treatment for Eurhadina (Eurhadina) dashuensis

XML Treatment for Eurhadina (Singhardina)

## Appendix 1

**Checklist of *Eurhadina* Haupt, 1929 from China**

**Eurhadina (Eurhadina) Haupt, 1929**

**E. (E.) alba Dworakowska, 1979**

E. (E.) alba Dworakowska, 1979: 709.

Distribution: China (Hunan); Japan.

**E. (E.) callissima Dworakowska, 1967**

*E. callissima* Dworakowska, 1967: 633, figs 14, 15.

E. (E.) callissima, Dworakowska 1982: 157.

Distribution: China (Shaanxi); Mongolia; Russia.

**E. (E.) cervina Tang, sp. nov**.

Distribution: China (Anhui).

**E. (E.) dashuensis Tang, sp. nov**.

Distribution: China (Anhui).

**E. (E.) fujiana Yang & Li, 1991**

E. (E.) fujiana Yang & Li, 1991: 27, figs 38–42.

Distribution: China (Fujian).

**E. (E.) japonica Dworakowska, 1971**

E. (E.) japonica Dworakowska, 1971: 652, figs 22–24.

Distribution: China (Anhui, Guizhou, Shaanxi, Sichuan); Japan.

**E. (E.) pulchella (Fallén, 1806)**

*Cicada pulchella* Fallén, 1806: 36.

*Typhlocyba pulchella*: Herrich-Schäffer 1835: 67.

*Cicadula pulchella*: Zetterstedt 1837: 301.

*Eupteryx ornatipennis* Curtis, 1837: 221. Synonymised by Dohrn 1859: 86.

*Typhlocyba lutea* Hardy, 1850: 418. Synonymised by Dohrn 1859: 86.

*Typhlocyba aurantiaca* Snellen van Vollenhoven, 1862: 85. Synonymised by Fokker 1891: 376.

*Eupteryx pulchellus*: Marshall 1867: 200.

*E. pulchella*: Haupt 1929: 1075.

Eupteryx (Eurhadina) pulchella: Ossiannilsson 1938: 75.

Cicadella (Eurhadina) pulchella: Kloet and Hincks 1945: 56.

E. (E.) pulchella pulchella: Dworakowska 1969: 72, figs 1–5, 13–15, 49, 63, 85.

E. (E.) pulchella orientalis Dworakowska, 1969: 74, figs 6, 16–18, 42, 64, 86. Synonymised by Nast 1972: 287.

Distribution: Algeria; China (Hunan, Shaanxi, Shandong); Europe; Japan; Kazakhstan; North Korea; Russia.

**Eurhadina (Singhardina) Mahmood, 1967**

**Eurhadina (Singhardina) rubra species group (Zhou et al. 2022)**

**E. (S.) foliiformis Zhang & Huang, 2022**

E. (S.) foliiformis Zhang & Huang in Zhou et al. 2022: 6, fig. 1.

Distribution: China (Yunnan).

**E. (S.) galacta Zhang & Huang, 2022**

E. (S.) galacta Zhang & Huang in Zhou et al. 2022: 8, fig. 2.

Distribution: China (Yunnan).

**E. (S.) menglunensis Huang & Zhang, 1999**

*E. menglunensis* Huang & Zhang, 1999: 252, fig. 6.

E. (S.) menglunensis: Zhou et al. 2022: 10, fig. 3.

Distribution: China (Yunnan).

**E. (S.) recta Zhang & Huang, 2022**

E. (S.) recta Zhang & Huang in Zhou et al. 2022: 12, fig. 4.

Distribution: China (Yunnan).

**E. (S.) rubra Dworakowska, 1969**

E. (S.) rubra Dworakowska, 1969: 76, figs 22, 23, 33, 40, 47, 52, 57, 91, 96, 105; Dworakowska 2002: 58, figs 92–100.

Distribution: China (Anhui, Fujian, Hunan, Yunnan); Malaysia; Vietnam.

**E. (S.) rubrocorona Cai & Kuoh, 1993**

*E. rubrocorona* Cai & Kuoh, 1993: 223, fig. 2.

E. (S.) rubrocorona: Zhou et al. 2022: 15.

Distribution: China (Anhui, Fujian, Guizhou, Zhejiang).

**E. (S.) scalesa Zhang & Huang, 2022**

E. (S.) scalesa Zhang & Huang in Zhou et al. 2022: 15, fig. 6.

Distribution: China (Yunnan).

**E. (S.) scamba Zhang & Huang, 2022**

E. (S.) scamba Zhang & Huang in Zhou et al. 2022: 17, fig. 7.

Distribution: China (Yunnan).

**E. (S.) scandens Zhang & Huang, 2022**

E. (S.) scandens Zhang & Huang in Zhou et al. 2022: 19, fig. 8.

Distribution: China (Yunnan).

**E. (S.) unipunicea Hu & Kuoh, 1991**

*E. unipunicea* Hu & Kuoh, 1991: 259, fig. 5.

E. (S.) unipunctata: Zhou et al. 2022: 21 (misspelling of *E. unipunicea*).

Distribution: China (Anhui, Yunnan).

**Eurhadina (Singhardina) vittata species group (Dworakowska 2002)**

**E. (S.) amacularis Zhang & Huang, 2022**

E. (S.) amacularis Zhang & Huang in Zhou et al. 2022: 21, fig. 9.

Distribution: China (Yunnan).

**E. (S.) parilintanonica Zhang & Huang, 2022**

E. (S.) parilintanonica Zhang & Huang in Zhou et al. 2022: 23, fig. 10.

Distribution: China (Yunnan).

**Eurhadina (Singhardina) punjabensis species group (Dworakowska 2002)**

**E. (S.) anurous Zhang & Xiao, 2000**

E. (S.) anurous Zhang & Xiao, 2000: 110, figs 15–26.

Distribution: China (Yunnan).

**E. (S.) biavis Yang & Li, 1991**

E. (S.) biavis Yang & Li, 1991: 25, figs 13–18.

*E. rubromia* Cai & Kuoh, 1993: 225, fig. 4. Synonymised by Zhou et al. 2022: 26, fig. 11.

Distribution: China (Anhui, Fujian, Guangxi, Yunnan, Zhejiang).

**E. (S.) centralis Yang & Li, 1991**

E. (S.) centralis Yang & Li, 1991: 26, figs 25–31.

Distribution: China (Fujian, Hunan, Zhejiang).

**E. (S.) choui Huang & Zhang, 1999**

*E. choui* Huang & Zhang, 1999: 247, fig. 2.

E. (S.) choui: Dworakowska 2002: 57.

Distribution: China (Fujian, Hunan).

**E. (S.) cuii Huang & Zhang, 1999**

*E. cuii* Huang & Zhang, 1999: 251, fig. 5.

E. (S.) cuii: Dworakowska 2002: 62.

Distribution: China (Fujian).

**E. (S.) dazhulana Yang & Li, 1991**

E. (S.) dazhulana Yang & Li, 1991: 23, figs 1–6.

Distribution: China (Anhui, Fujian, Hunan, Yunnan, Zhejiang).

**E. (S.) diplopunctata Huang & Zhang, 1999**

*E. diplopunctata* Huang & Zhang, 1999: 253, fig. 7.

E. (S.) diplopunctata: Zhou et al. 2022: 28, fig. 13.

Distribution: China (Anhui, Fujian, Hunan, Yunnan, Zhejiang).

**E. (S.) exclamationis Yang & Li, 1991**

E. (S.) exclamationis Yang & Li, 1991: 23, figs 7–12; Dworakowska 2002: 62.

Distribution: China (Anhui, Fujian).

**E. (S.) extensa Zhang & Huang, 2022**

E. (S.) extensa Zhang & Huang in Zhou et al. 2022: 30, fig. 14.

Distribution: China (Yunnan).

**E. (S.) fasciata Dworakowska, 2002**

E. (S.) fasciata Dworakowska, 2002: 56, figs 73–82.

Distribution: China (Jiangxi, Yunnan); Thailand.

**E. (S.) flavicorona Cai & Kuoh, 1993**

*E. flavicorona* Cai & Kuoh, 1993: 224, fig. 3.

E. (S.) flavicorona: Dworakowska 2002: 57.

Distribution: China (Anhui, Fujian, Hunan).

**E. (S.) flaviscutella Zhang & Huang, 2022**

E. (S.) flaviscutella Zhang & Huang in Zhou et al. 2022: 34, fig. 16.

Distribution: China (Anhui, Fujian).

**E. (S.) fusca Cai & Kuoh, 1993**

*E. fusca* Cai & Kuoh, 1993: 222, fig. 1.

E. (S.) fusca: Dworakowska 2002: 47.

Distribution: China (Fujian, Yunnan).

**E. (S.) gracilifurca Zhang & Huang, 2022**

E. (S.) gracilifurca Zhang & Huang in Zhou et al. 2022: 36, fig. 17.

Distribution: China (Yunnan).

**E. (S.) lata Zhang & Huang, 2022**

E. (S.) lata Zhang & Huang in Zhou et al. 2022: 38, fig. 18.

Distribution: China (Yunnan).

**E. (S.) nasti Dworakowska, 1969**

E. (S.) nasti Dworakowska, 1969: 76, figs 29–31, 37, 38, 45, 53, 56, 97, 98, 106.

Distribution: China (Guangdong).

**E. (S.) rubrania Huang & Zhang, 1999**

*E. rubrania* Huang & Zhang, 1999: 246, fig. 1.

E. (S.) rubrania: Dworakowska 2002: 66.

Distribution: China (Hunan, Zhejiang).

**E. (S.) rubrivittata Chiang, Hsu & Knight, 1989**

E. (S.) rubrivittata Chiang, Hsu & Knight, 1989: 125, fig. 14.

Distribution: China (Taiwan).

**E. (S.) rutilans Hu & Kuoh, 1991**

*E. rutilans* Hu & Kuoh, 1991: 260, fig. 6.

E. (S.) rutilans: Dworakowska 2002: 56.

Distribution: China (Anhui, Yunnan); Thailand.

**E. (S.) spinifera Huang & Zhang, 1999**

*E. spinifera* Huang & Zhang, 1999: 248, fig. 3.

E. (S.) spinifera: Zhou et al. 2022: 42, fig. 20.

Distribution: China (Fujian).

**E. (S.) tripunctata Huang & Zhang, 1999**

*E. tripunctata* Huang & Zhang, 1999: 254, fig. 8.

E. (S.) tripunctata: Dworakowska 2002: 68.

Distribution: China (Fujian).

**E. (S.) unilobata Chiang, Hsu & Knight, 1989**

E. (S.) unilobata Chiang, Hsu & Knight, 1989: 125; Dworakowska 2002: 94.

Distribution: China (Taiwan).

**E. (S.) uprotrusa Zhang & Huang, 2022**

E. (S.) uprotrusa Zhang & Huang in Zhou et al. 2022: 43, fig. 21.

Distribution: China (Yunnan).

**E. (S.) wuyiana Yang & Li, 1991**

E. (S.) wuyiana Yang & Li, 1991: 26, figs 19–24.

*E. flavescens* Huang & Zhang, 1999: 250, fig. 4. Synonymised by Zhou et al. 2022: 45, fig. 22.

Distribution: China (Fujian, Hunan, Sichuan, Yunnan, Zhejiang).

**E. (S.) zadyma Dworakowska, 2002**

E. (S.) zadyma Dworakowska, 2002: 49, figs 18–27.

Distribution: China (Yunnan); Thailand.

**Eurhadina (Singhardina) mamata species group (Dworakowska 2002)**

**E. (S.) acapitata Dworakowska, 1982**

*E. acapitata* Dworakowska, 1982: 159, figs 687–693.

E. (S.) acapitata: Dworakowska 2002: 79.

Distribution: China (Taiwan).

**E. (S.) flavistriata Yang & Li, 1991**

E. (S.) flavistriata Yang & Li, 1991: 27, figs 32–37.

Distribution: China (Anhui, Fujian, Zhejiang).

**E. (S.) jarrayi Dworakowska, 2002**

E. (S.) jarrayi Dworakowska, 2002: 72, figs 180–188.

Distribution: China (Yunnan); Thailand.

**E. (S.) prima Dworakowska, 2002**

E. (S.) prima Dworakowska, 2002: 81, figs 248–258.

Distribution: China (Yunnan); Thailand.

**E. (S.) quadrimacularis Zhang & Huang, 2022**

E. (S.) quadrimacularis Zhang & Huang in Zhou et al. 2022: 52, fig. 26.

Distribution: China (Guangxi, Yunnan).

**E. (S.) yingfengica Dworakowska, 2002**

E. (S.) yingfengica Dworakowska, 2002: 77, figs 223–234.

Distribution: China (Taiwan, Yunnan).

**Eurhadina (Singhardina) robusta species group (Dworakowska 2002)**

**E. (S.) dissimilis Zhang & Huang, 2021**

E. (S.) dissimilis Zhang & Huang, 2021: 512, figs 26–38.

Distribution: China (Yunnan).

**E. (S.) flatilis Zhang & Huang, 2021**

E. (S.) flatilis Zhang & Huang, 2021: 512, figs 39–51.

Distribution: China (Yunnan).

**E. (S.) fumosa Zhang & Huang, 2021**

E. (S.) fumosa Zhang & Huang, 2021: 513, figs 52–64.

Distribution: China (Yunnan).

**E. (S.) furca Zhang & Huang, 2021**

E. (S.) furca Zhang & Huang, 2021: 519, figs 65–77.

Distribution: China (Yunnan).

**E. (S.) immatura Zhang & Huang, 2021**

E. (S.) immatura Zhang & Huang, 2021: 519, figs 78–90.

Distribution: China (Yunnan).

**E. (S.) krispinilla Dworakowska, 2002**

E. (S.) krispinilla Dworakowska, 2002: 87, figs 303–310; Zhou et al. 2021: 512, figs 1–13.

Distribution: China (Yunnan); Thailand.

**E. (S.) pookiewica Dworakowska, 2002**

E. (S.) pookiewica Dworakowska, 2002: 91, figs 335–344; Zhou et al. 2021: 512, figs 14–25.

Distribution: China (Yunnan); Thailand.

**Eurhadina (Zhihadina) Yang & Li, 1991 (*nomen dubium* )**

**E. (Z.) sinica Yang & Li, 1991**

E. (Z.) sinica Yang & Li, 1991: 29 (*nomen dubium* and *incertae sedis*).

Distribution: China.

## Additional information

### Artificial Intelligence (AI) use

The authors accept full responsibility for the content of the manuscript, including the disclosure of any use of AI.

No AI tools were used in the preparation of this manuscript.

### Author ORCIDs

X. Huang https://orcid.org/0009-0001-1888-5615

J. Wang https://orcid.org/0009-0001-0914-492X

L. Tang https://orcid.org/0009-0008-7518-0751

M.D. Webb https://orcid.org/0000-0002-1312-6142

Y. Duan https://orcid.org/0000-0002-5952-0778

### Data availability

All of the data that support the findings of this study are available in the main text.
